# Wheel Force Sensor-Based Techniques for Wear Detection and Analysis of a Special Road

**DOI:** 10.3390/s18082493

**Published:** 2018-08-01

**Authors:** Huawen Yan, Weigong Zhang, Dong Wang

**Affiliations:** School of Instrument Science and Engineering, Southeast University, Nanjing 210096, China; keep999@seu.edu.cn

**Keywords:** wheel force sensor, WFS, automobile proving ground, special road, dynamic detection

## Abstract

Automobile proving ground is important for the research of vehicles which are used for vehicle dynamics, durability testing, braking testing, etc. However, the road in automobile proving grounds will inevitably be damaged with the extension of the service life. In most previous research, equipment similar to a laser profilometer was used to detect the quality of the road, the principle of which is to reflect the quality of the road by measuring the roughness of the pavement. This method ignores the elastic deformation of the road itself when the vehicle is traveling and it is difficult to compensate for the error. Therefore, this paper presents a new method based on a force sensor to reduce the impact of elastic deformation, such as tire deformation, pavement deformation, and wheel rim deformation. In this study, force sensors mounted on the wheels collect the three-dimensional dynamic force of the wheel. The presented method has been tested with two sets of cobblestone road loads, and the result shows that the load intensities imposed by the test vehicle on the target road are 88.3%, 91.0%, and 92.05% of the intensity of the load imposed by the test vehicle on a standard road in three respective dimensions. It is clear that the proposed method has strong potential effectiveness to be applied for wear detection and analysis of a special road.

## 1. Introduction

A prototype test is a key part to develop a new car, which is responsible for detecting the potential defects in the previous part and for testing some of the important features of the car, such as power, reliability, ride comfort, handling and durability. For example, reference [1] proposes a new anti-lock braking system (ABS) for electric vehicles using a virtual load, and a sliding-mode controller is proposed for regulating the slip ratio to reach the ideal value for road adhesion; Reference [2] presents a detection method for damage location of beam structures based on the mode shape extracted from the dynamic load of the vehicle to reduce the impact of the external environment; the presented method has been tested with experiments, and the results show that the accuracy of location can exceed 97%; The road load is widely used in automotive durability testing; in order to achieve the durability of the entire vehicle, it is necessary for the entire vehicle, system, subsystem and parts to meet their respective durability requirements [3]; Automobile operational stability is also closely related to the road load, the main test items are: a low-speed steering and portability test, steady-state steering characteristic test, transient yaw response test, automotive return-to-negative capability test, steering wheel angle pulse test, steering wheel intermediate position manipulation stability test, etc. [4]; A key factor in the test of automobile fatigue life is accurate acquisition of the random load imposed on the tested item [5]. Mass production is impossible unless the prototype test has achieved complete success.

**Figure 1:**
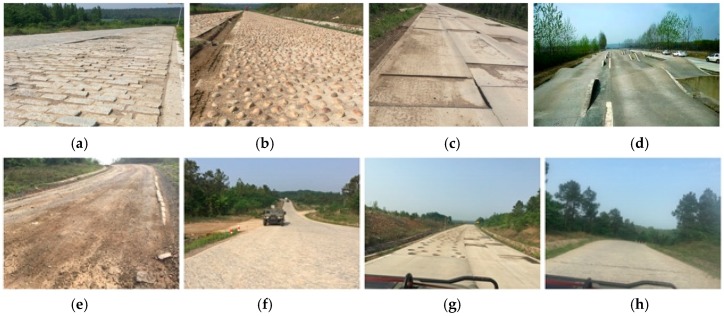
The special roads: (**a**) Belgium road; (**b**) Cobblestone road; (**c**) Square pit road; (**d**) Twisted road; (**e**) Muddy road; (**f**) Gravel road; (**g**) Round pit road; (**h**) Stone road.

Tests on special roads of automobile proving ground are an important part of the prototype test, including the cobblestone road test, wash plate road test, gravel road test, wave road test, Belgium road test, twisted road test, pit road test, stone road test, muddy road test and so on, some of the special roads are shown in Figure 1. Different special roads result in vehicle loads of different frequencies and different intensities, and focus is on performance testing from different perspectives; the cobblestone road as shown in Figure 1b is the key research object of this paper. With the extension of road service life, the road will inevitably be damaged. Traditionally, equipment such as a level meter-bar, a multi-wheel measuring horizontal vehicle, an accumulative jolt instrument, a TML high speed pavement meter, a GMR pavement meter, and a laser profilometer is used to evaluate the quality of the road.

The principle of the level meter-bar is to establish a fixed and non-vibrating reference and to measure the deviation between the road surface and the reference [6]. The principle of a multi-wheel measuring horizontal vehicle is similar to that of a level meter-bar, but its reference is mounted on the supporting wheel of a multi-wheel measuring horizontal vehicle to achieve continuous measurement [7]. The accumulative jolt instrument is a mechanical vibration system that runs on the measured road at a certain speed, and its measurement method is indirect measurement [8]. The TML high-speed pavement meter is used to find the next road surface data by a recursive method based on the known road surface data and measurement parameters [9]. The GMR pavement meter based on the inertial reference can directly measure the height difference of the road surface [10]. The laser profilometer directly measures the road profile by a laser sensor installed in front of the vehicle [11]. According to all the above documents, their core principle is to evaluate the road by pavement roughness. Those methods assessing the road grade by road traffic department ignore the road and vehicle interaction process, because the road itself will produce elastic deformation while the vehicles pass it, and the deformation affects the accuracy of the roughness. We know that the purpose of special roads is to provide sufficient intensities of load to speed up the testing of vehicles, which is essentially different from the road quality inspection of the road rating department, especially in the evaluation of special roads. There are inherent deficiencies in the methods adopted by the road evaluation department. Therefore, this paper presents a new evaluation method based on wheel force sensor (WFS), which can accurately obtain the loads applied to the road by the vehicle, and then evaluate the road quality by the statistical analysis of the loads. This approach takes the interaction between the vehicle and the road into account, which is more comprehensive than the methods of evaluating the road’s geometric dimensions alone. WFS is a high-accuracy, 6-axis wheel force measurement system for real-time measurement of forces (F*x*, F*y*, F*z*) and moments (M*x*, M*y*, M*z*) acting on the wheel hub under dynamic conditions [12]. Currently, some commercial WFS are available, including the 5400 series multiple-axis WFS (PCB Group), the LW series six-dimensional WFS (Kistler Group, Winterthur, Switzerland), SWIFT (MTS Systems Corporation, Eden Prairie, ME, USA) and the SLW-NC WFS (Tokyo Sokki Kenkyojo Co., Ltd., Tokyo, Japan). They have all been applied in many vehicle applications and have acceptable performances [13]; however, the application of WFS in road evaluation has rarely occurred. This paper uses a three-dimensional force sensor acquisition system developed by Southeast University to achieve wear detection and analysis of a special road, which has a pioneering significance and aims to overcome the problem of dynamic distortion that traditional methods cannot avoid. The WFS is mounted on the axle and rotates with the wheel to dynamically measure the load applied to the road by the vehicle, and the multi-dimensional force sensor measures the force of the wheel in three dimensions.

## 2. Materials and Methods

### 2.1. The Principle of Road Load Acquisition

**Figure 2:**
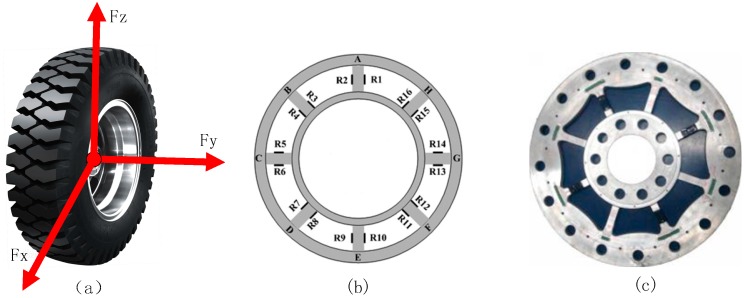
(**a**) Force diagram; (**b**) Diagram of the strain gauge arrangement; (**c**) Wheel force sensor.

When a car runs on a road, the forces on the three dimensions are applied to the wheel because of the interaction between the wheel and the road, namely, the longitudinal force F*x*, the lateral force F*y* and the vertical force F*z* [14], as shown in Figure 2a. It is generally known that the effect of force is mutual, so the force applied to the wheel is equivalent to the road load, in other words, the three-dimensional forces of the wheel are equal to the three-dimensional dynamic loads of the road. The WFS is attached to the wheel and rotates with it in order to collect the dynamic load of the wheel. The core principle of WFS is using the strain gauge elastic beams to reflect the road load. Figure 2b,c are the structural and physical views of the 8-beam WFS, respectively. The maximum non-linearity error, the maximum hysteresis error, the maximum repeatability error of the proposed WFS are 0.9% of the full scale, 1.1% of the full scale and 0.5% of the full scale, respectively [14].

### 2.2. Road Load Acquisition System

**Figure 3:**
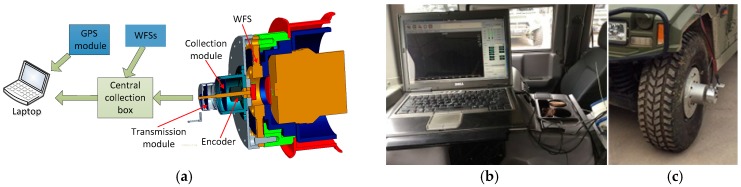
(**a**) The diagram of the WFS System; (**b**) PC processing end; (**c**) data acquisition end.

The WFS road load acquisition system is a platform that combines the wheel force sensor and a vehicle. Its block diagram is shown in Figure 3a–c, indicating the PC processing end and data acquisition end. The hardware components of the system include WFS, collection module, encoder, transmission module, central collection box, GPS module and laptop. The WFS is connected to the hub of the wheel by customizing the flange plate and enables the collection of road loads in the form of elastic deformation. When the vehicle runs on a road, the wheel and the WFS connected to it simultaneously perform rotational and translational motions, while the body of the car only performs translational motion, which brings two problems: The first is how to send the elastic deformation signal collected by the rotating WFS to the PC processing end in the car stably and quickly; the second is how to separate highly coupled *x*-axis signals and *z*-axis signals.

In order to solve these two problems, a Bluetooth wireless transmission module and an angle encoder were introduced. The Bluetooth wireless module is responsible for transmitting the rotating WFS signal to the relatively stationary PC processing end, while the angle encoder is responsible for solving the problems of the *x*-axis and *z*-axis interaction. The WFS signals and angle signals of each wheel are sent to the central collection box through the collection module and transmission module and are finally sent to the PC processing end through a network cable. In the upper computer software, the WFS signal and the GPS vehicle speed signal are further analyzed, processed and displayed synchronously by the data analysis software.

**Figure 4:**
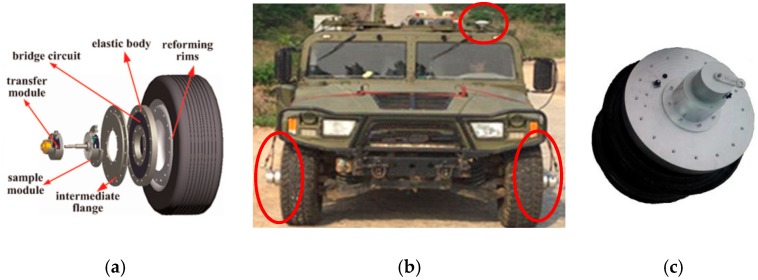
(**a**) The diagram of the tire with WFS, (**b**) The vehicle with WFS system, (**c**) The rim with WFS.

The WFS system is a vehicle-to-road coupling system, so the measured loads are related to multiple factors, which include road type, vehicle type, speed, tire pressure and so on. The road load of different roads is not the same, which is the reason for setting various special roads. Different vehicles have different suspension types and weights, and their loads on the road are naturally different. In addition, the impact of speed and tire pressure is also evident. The vehicle used in this experiment is a four-wheel-drive military vehicle, with a total weight is equal to 3.7 t including curb weight, weight of testers and equipment; the tire pressure was adjusted to 2.65 bar, and the vehicle with WFS system and the rim with WFS are shown in Figure 4.

**Figure 5:**
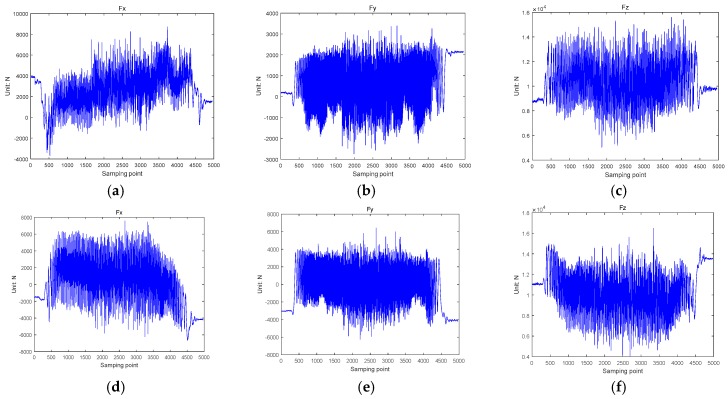
The original loads on the standard road, (**a**) F*x* of the left WFS, (**b**) F*y* of the left WFS, (**c**) F*z* of the left WFS, (**d**) F*x* of the right WFS, (**e**) F*y* of the right WFS, (**f**) F*z* of the right WFS.

The sensors in the WFS system are mounted on the left and right wheels of the front axle. The special road used in the experiment is a cobblestone road, which includes a standard road and a target road. The speed of the vehicle is maintained at 40 km/h. The original loads collected by the WFS on the standard road are shown in Figure 5a–c, which represent the three dimensional loads F*x*, F*y*, and F*z* of the left WFS, and Figure 5d–f represent the three dimensional loads F*x*, F*y*, and F*z* of the right WFS. The sensor collects a set of data every 5 ms that is, the sampling rate is 200 Hz. The length of the standard cobblestone road is 200 m. Approximately 5000 effective points were collected when the test vehicle passed at a speed of 40 km/h. From Figure 5a,d, it can be seen that the driving force (F*x*) is about 2000 N. From Figure 5b,e, it can be seen that the lateral force (F*y*) is about 0 N. From Figure 5e,f, it can be seen that the vertical force (F*z*) is about 10 KN.

**Figure 6:**
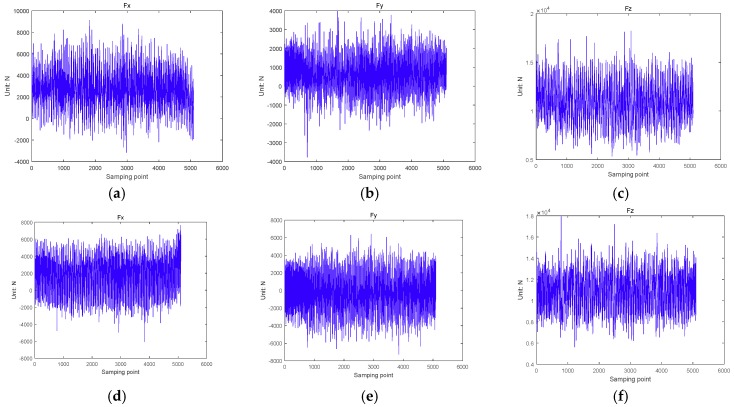
The original loads on the target road, (**a**) F*x* of the left WFS, (**b**) F*y* of the left WFS, (**c**) F*z* of the left WFS, (**d**) F*x* of the right WFS, (**e**) F*y* of the right WFS, (**f**) F*z* of the right WFS.

The original loads collected by the WFS on the target road are shown in Figure 6. Similar to Figure 5, Figure 6a–c represents the three-dimensional loads F*x*, F*y*, and F*z* of the left WFS, and Figure 6d–f represents the three-dimensional loads F*x*, F*y*, and F*z* of the right WFS. The target cobblestone road is also 200 m long. Similar to the standard road, approximately 5000 effective points are collected. From Figure 6a,d, it can be seen that the driving force F*x* is about 2000 N. From Figure 6b,e, it can be seen that the lateral force F*y* is about 0 N. From Figure 6e,f, it can be seen that the vertical force F*z* is about 10,000 N. Comparing Figure 5 and Figure 6, the data of the three-dimensional load have a small difference, and it is difficult to find the essential differences between the two roads. It is not easy to distinguish the difference between the original loads of standard road and the original loads of the target road from the curve form, and it is difficult to quantify the damage degree of the target road. However, if these intensities of load are quantified by suitable methods, it will easy to see the degree of wear of the target road. The suitable method used in this paper is the rain flow-counting method.

### 2.3. Road Load Processing for Rain flow Counting Method

**Figure 7:**
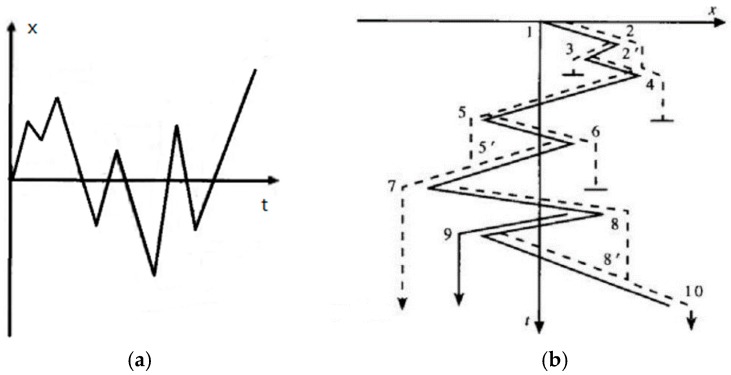
Rain flow counting principle schematic diagram. (**a**) A sequence of random load data, (**b**) Clockwise rotation of 90 degrees of random load data.

In order to cope with the quantification problem, a widely used method in the fatigue life calculation rain flow counting method is introduced. The rain flow counting method is used in the analysis of fatigue data in order to reduce a spectrum of varying stress into a set of simple stress reversals [15]. Its importance is that it allows the application of Miner’s rule in order to assess the fatigue life of a structure subject to complex loading [16]. The process of counting the load by the rain flow counting method reflects the memory characteristics of the material and has a clear mechanical concept, so the method has been widely used [17]. The basic counting rules are as follows:

The rain flows down the slope from the inside of the peak (valley) value of the load-time series;

A: Flow stops when the absolute value of peak (valley) value is greater than that of the initial peak (valley) value;

B: Flow stops when encountering the above-mentioned rain;

Remove all of the full cycles and writing down the valley and peak values for each cycle;

The first stage of the counting will leave a divergent convergence load-time series; convert it into a convergent-divergence of the load-time series, and then start the second stage of the counting until the all of the loads are counted. An example is shown in Figure 7.

A random load sequence is shown in Figure 7a. The first step is to rotate it clockwise 90 degrees to get Figure 7b. The rain flow method starts at tensile peak 1, which is considered to be the minimum value. The rain flows to peak 2, drops vertically to 2′ between tensile peak 3 and 4, then flows to tensile peak 4, and finally stops at the corresponding point of more negative peak 5 than earlier peak 1, producing half-cycles from 1 to 4. The next rain flow begins at peak 2 and flows through 3, stopping at the opposite of 4, because 4 is a greater maximum than the starting 2, resulting in a half-cycle 2–3. The third flow starts at peak 3, because it encounters the rain flow from 2, so it ends at 2′, resulting in a half cycle of 3–2′. In this way, 3–2 and 2–3 form a closed stress-strain loop, that is configured as a complete loop 2′–3–2. The 4th flow starts from peak 4 and passes through peak 5, dropping vertically to 5′ between 6 and 7, continuing to flow downwards, and then dropping from 7 vertically to the opposite of peak 10, since peak 10 has a greater maximum value than peak 4; half cycles 4–5–7 are derived. The 5th flow starts from peak 5, flows to peak 6, drops vertically, and ends at the opposite of 7, because peak 7 has more negative minima than peak 5. Then, take out half cycle 5–6. The 6th flow starts at peak 6 and ends at 5′ because it encounters raindrops from the peak 5 outflow. Half-cycles 6–5 and 5–6 are combined into a complete cycle of 5′–6–5. The 7th flow starts at peak 7 and goes through peak 8 and falls to the 8′ on the 9–10 line. Then, it goes to the last peak 10 and takes out the semicircle 7–8–10. The 8th flow starts from peak 8 and ends on the opposite side where the flow drops to 9 down to 10 because peak 10 has a more positive value than 8 and half cycles 8–9 are taken out. The last flow started at a peak of 9 because it encountered a rain drop from a peak of 8, so it ended at 8′. Half-cycles 9–8′ are obtained and paired with 8–9 to form a complete cycle of 8–9–8′. Thus, the strain-time record shown in Figure 7b includes three full-cycle 8–9–8′, 2–3–2′, 5–6–5′, and three half-cycles 1–2–4, 4–5–7, 7–8–10 [18].

**Figure 8:**
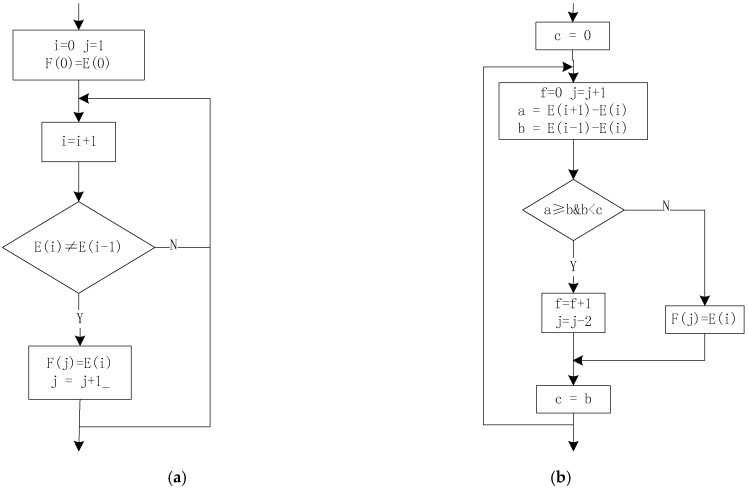
(**a**) Compression equivalent point, (**b**) Extract cycle diagram.

The rain flow-counting method is very suitable for computer programming based on the above counting rules. Figure 8 shows how the rain flow-counting method is implemented in the program. It includes two steps: data compression and cycle number extraction. The data compression program flow diagram is shown in Figure 8a. Set the array to be processed to E(n) and the resulting array to F(n). i and j are the numbers of two array elements, respectively. In the compression of adjacent equivalence numbers, the diamond’s judgment condition is whether the adjacent two elements are not equal. If true, the number is left, otherwise the judgment of the next number will continue until the last number. This removes the first number when it encounters an equal number. The rain flow counting is to extract the cycle from the compressed data and record its characteristic values such as peak, valley and amplitude. The four-point method that is easier to implement is used in the program. The specific implementation method is shown in flow Figure 8b. In the flow chart, the initial setting of c = 0 and the following c = b are some optimizations for the program. The four-point method actually uses only three points. This puts the last calculated E(i − 1) − E(i) as the current E(i − 1) − E(i − 2). s is a flag of whether or not there is a loop. It can be judged whether or not s is equal to 0 in Figure 8b. If it is equal to 0, it means that all the loop counts are obtained, otherwise, the entire process of Figure 8b will continue to be executed. s is the total number of recording cycles.

## 3. Results

**Figure 9:**
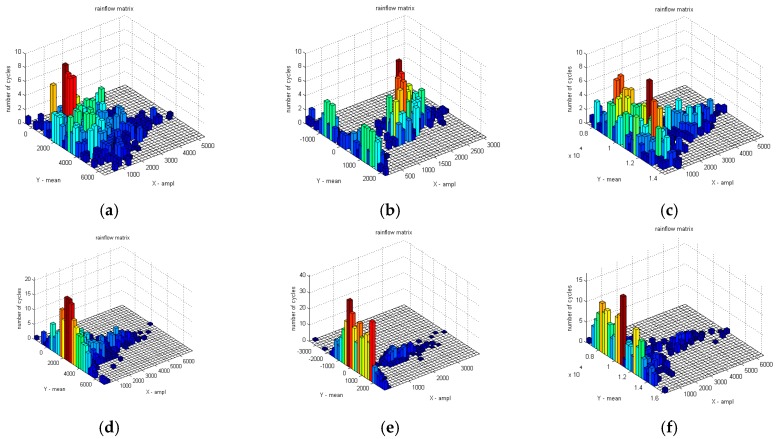
Rain flow matrix of load on the standard road. (**a**) Rain flow matrix of F*x* on the left WFS, (**b**) Rain flow matrix of F*y* on the left WFS, (**c**) Rain flow matrix of F*z* on the left WFS, (**d**) Rain flow matrix of F*x* on the right WFS, (**e**) Rain flow matrix of F*y* on the right WFS, (**f**) Rain flow matrix of F*z* on the right WFS.

**Figure 10:**
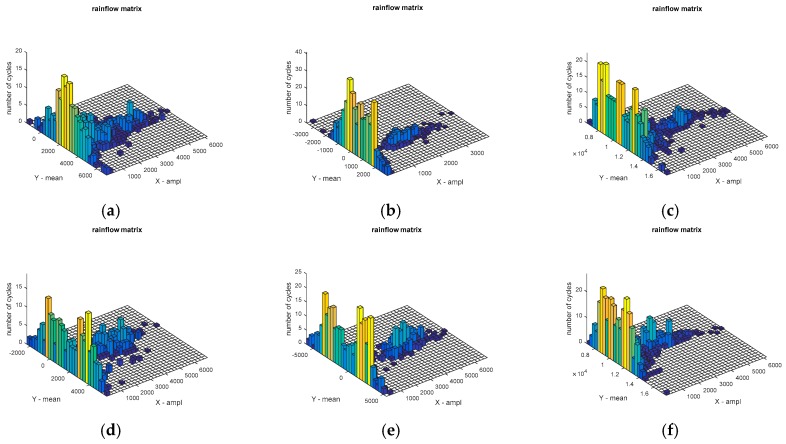
Rain flow matrix of load on the target road. (**a**) Rain flow matrix of F*x* on the left WFS, (**b**) Rain flow matrix of F*y* on the left WFS, (**c**) Rain flow matrix of F*z* on the left WFS, (**d**) Rain flow matrix of F*x* on the right WFS, (**e**) Rain flow matrix of F*y* on the right WFS, (**f**) Rain flow matrix of F*z* on the right WFS.

The original load data collected by the WFS are input to the rain flow counting program, and results of the rain flow matrix are shown in Figure 9 and Figure 10.

**Figure 11:**
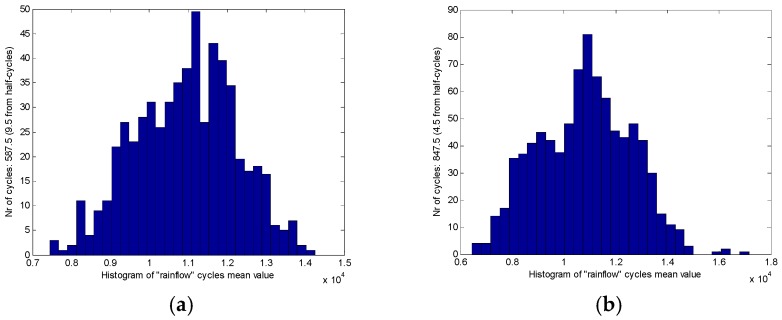
The mean value of the F*z*, (**a**) On the standard road, (**b**) On the target road.

**Figure 12:**
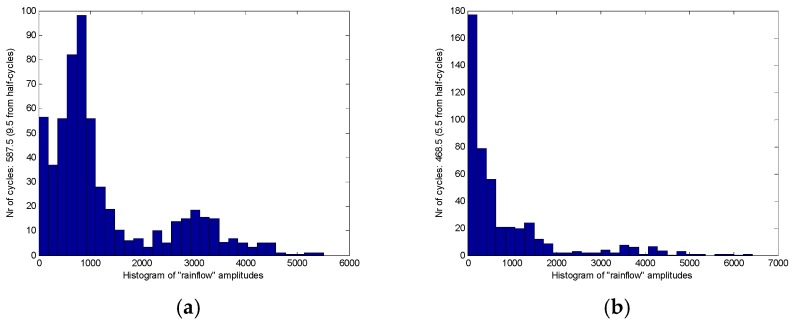
The amplitudes value of the F*z*, (**a**) On the standard road, (**b**) On the target road.

The analysis of the rain flow matrix of load on the target road (Figure 10) is similar to the standard road (Figure 9), which is also used in the original load data in the rain flow program for obtaining the corresponding rain flow matrix. The *x* and *y*-axes of the rain flow matrix are the cycle count of the amplitude and the cycle count of the mean, respectively. Through the comparison between Figure 9 and Figure 10, it can be clearly seen that the standard road can provide more intensities of load to the test vehicle than the target road. The reason is that the target road was worn out during the service. The road load can be quantitatively analyzed after the previous processing, which is also the core value of rain flow method. Figure 9a,d are the rain flow matrix of traction force F*x* of the WFS on the standard road. It can be seen from Figure 9a,d that the number of rain cycles of F*x* are mostly concentrated at about 2000 N, which is consistent with the original load in Figure 5a,d. However, specific quantitative data can be obtained in the rain flow matrix. Similarly, Figure 9b,e reflects that the number of lateral force rain flow cycles is mainly around 0 N, and Figure 9c,f reflects the number of vertical force rain cycles mainly concentrated near 1.1 KN, which also are consistent with the original load in Figure 5. In order to do a further description, it is possible to choose any side of WFS and the load of any dimension. So the vertical load (F*z*) on the left WFS was selected in this paper. The mean value of the F*z* of the standard cobblestone road and the target cobblestone road is shown as in Figure 11, and the amplitude values of the F*z* of the standard cobblestone road and the old cobblestone road are shown as in Figure 12. It can be seen from Figure 11 that the mean value of F*z* is mainly concentrated on a quarter of the car’s weight, and the effect on the standard cobblestone road and the target cobblestone road was not significantly different, which is consistent with the car weight. The amplitude value is obviously different from Figure 12. The amplitude values under 2000 N of the standard road and the old road are relatively abundant, while the number of load cycles between 2000 N to 4000 N is not the same. The number of load cycles of the target road is clearly less than the standard one, which indicates that the target road has worn out, and it hardly provides enough intensities of loads of 2000 N–4000 N.

A more detailed description is shown in Table 1, which is rain flow counts after quantification of each dimension load. The theory of fatigue damage accumulation states that when the stress on a part exceeds the fatigue limit, each load cycle causes a certain amount of damage to the part, and this damage can accumulate; when the damage accumulates to a critical value, the part will suffer fatigue failure. The linear fatigue damage accumulation theory holds that the fatigue damage generated by each cycle load is independent of each other, so the total damage is the linear accumulation of each fatigue damage [19]. According to the linear fatigue damage accumulation theory, the three dimensions of the WFS load can be arranged as follows: (1) Since the load with a small amplitude has no effect on the fatigue damage of the road, only the number of cycles of the load whose magnitude exceeds 1000 N is calculated in the rain flow matrix; (2) The number of mean cycles of driving force F*x* is mainly concentrated near 2000 N, so 1000 N to 3000 N is taken as the counting range; (3) The number of mean cycles of lateral force F*y* is mainly concentrated near 0 N, so −1000 N to 1000 N is taken as the counting range; (4) The number of mean cycles of vertical force F*z* is mainly concentrated near 1.1 KN, so 0.8 KN to 1.4 KN is taken as the counting range.

Mean and amplitude are two equally important parameters, and the effect of the same load amplitude at different mean levels on the road is not the same. Similarly, different load amplitudes at the same average level also have different effects on the road. Therefore, the number of cycles in the last column of Table 1, which is the number of cycles that satisfy both the mean range and the amplitude range, is the core data. Seen from the table, the effective count of the driving force F*x* of the right WFS on the standard road and the target road is 553 and 489, respectively, which means that the target road has less impact on the test vehicle than on the standard road. It is easy to calculate the ratio $\sigma_{Fx\_R}$ between two quantified F*x*’s, which is equal to 88.4%. Similarly, the following 5 ratios can also be easily obtained, including $\sigma_{Fx\_L}=88.2\%$, $\sigma_{Fy\_R}=90.9\%$, $\sigma_{Fy\_L}=91.1\%$, $\sigma_{Fz\_R}=91.9\%$ and $\sigma_{Fz\_L}=92.2\%$. Therefore, after simply averaging the left WFS and right WFS, we can get the ratio of the standard road and the target road in three dimensions as shown in Equations (1)–(3).

(1) $$\sigma_{Fx}=\frac{\sigma_{Fx\_R}+\sigma_{Fx\_L}}{2}=88.3\%$$

(2) $$\sigma_{Fy}=\frac{\sigma_{Fy\_R}+\sigma_{Fy\_L}}{2}=91.0\%$$

(3) $$\sigma_{Fz}=\frac{\sigma_{Fz\_R}+\sigma_{Fz\_L}}{2}=92.05\%$$

## 4. Discussion

According to the existing literature of wheel force sensor application, researchers are concerned more about the research of WFS in the automotive industry, such as dynamics, durability and braking force studies. However, few people are involved in the application of WFS in road evaluation. In particular, WFS has a unique advantage in the dynamic evaluation of roads, as compared to traditional road surface roughness evaluation methods.

Based on the above analysis, we present several ways to promote the performance of wear detection and analysis of the special road in the further study: (1) Multiple road load collection experiment can increase the number of data sources to improve the stability and reliability of the test; (2) Improved the rain flow counting model making the calculation process more concise and convenient.

### 4.1. Multiple Road Load Collection Experiment

It is generally considered that the load intensity imposed by the special road on the test vehicle is a stationary random process. Therefore, it is only necessary to collect a set of load data to evaluate a special road according to the ergodic theory of stationary stochastic processes. However, there are many factors that affect the accuracy of the load data and cannot be prepared in advance, such as weather, ground humidity and temperature, etc. The load data used in this article were obtained on a single day. Therefore, it is hoped that multiple seasonal load data can be collected in the future experiment to eliminate the influence factors mentioned above and make the data as accurate and stable as possible. In addition, there are all kinds of special roads in the automobile testing grounds, up to 13 kinds. This article only discusses a special type of cobblestone road, and automobile testing will generally be a combination of several special types of road testing, Therefore, in the subsequent experiments, all other 12 roads can be assessed to complete the special road wear detection and analysis of the entire automobile proving ground.

### 4.2. The Rain Flow Counting Model Improvement

The four-peak-valley counting model used in this paper is a traditional calculation model that strictly according to the principle of rain flow counting method. It is the most widely used in practical projects currently. It generally includes the following 4 steps: (1) data compression; (2) first rain flow count; (3) closure processing of divergence-convergence data; (4) second rain flow count. It can be seen that the four-peak valley counting model contains two counts of rain flow, and the calculation is complicated. The three-peak-valley count model is based on the four-peak valley count model and is an improvement of the four-peak valley count model. It inherits the basic assumptions such as the closure of the four-peak-valley counting model and its independence from the loading sequence. The three-peak valley counting model guarantees the overall convergence and divergence of data by pre-processing the compressed data, that is, truncating and docking the compressed data. Therefore, only one rain flow counting is required. Simplifying the calculation process is very critical in real-time processing. This will fully prepare the WFS system for real-time evaluation of roads based on mobile platforms.

## 5. Conclusions

In order to overcome the shortcomings of traditional methods that cannot dynamically evaluate the special roads, a new method of road wear analysis based on WFS is proposed. After completing the assessment of the road with the WFS system, the loads of the special road for each wheel can be quantified. It is clear to know how many loads the standard road or the target road can provide to the vehicle, and after calculation, we know that the target road provides traction to the vehicle equivalent to 88.3% of that provided by the standard road, and 90.0% and 92.05% of the lateral and vertical forces, respectively. This is a way to analyze the quality of the road from the perspective of the purpose of the special roads, and it reflects the nature of special roads.

## Figures and Tables

**Table 1:** Rain flow counts after quantification of each dimension load.

			Mean Range	The Number of Mean Cycles	Amplitude Range	The Number of Amplitude Cycles	The Number of Cycles That Satisfy Both the Mean Range and the Amplitude Range
The standard road	The right WFS	F*x*	1000 N–3000 N	836	>1000 N	775	553
		F*y*	−1000 N–1000 N	845	>1000 N	712	587
		F*z*	8 KN–14 KN	1205	>1000 N	1031	844
	The left WFS	F*x*	1000 N–3000 N	822	>1000 N	745	576
		F*y*	−1000 N–1000 N	876	>1000 N	732	565
		F*z*	8 KN–14 KN	1188	>1000 N	989	812
The target road	The right WFS	F*x*	1000 N–3000 N	755	>1000 N	703	489
		F*y*	−1000 N–1000 N	765	>1000 N	683	534
		F*z*	8 KN–14 KN	1033	>1000 N	897	776
	The left WFS	F*x*	1000 N–3000 N	769	>1000 N	715	508
		F*y*	−1000 N–1000 N	778	>1000 N	682	515
		F*z*	8 KN–14 KN	994	>1000 N	917	749
